# Implementation of the BERT-derived architectures to tackle disinformation challenges

**DOI:** 10.1007/s00521-021-06276-0

**Published:** 2021-07-22

**Authors:** Sebastian Kula, Rafał Kozik, Michał Choraś

**Affiliations:** 1 UTP University of Science and Technology, Kaliskiego 7, 85-976 Bydgoszcz, Poland; 2 Kazimierz Wielki University UKW, Bydgoszcz, Poland

**Keywords:** Fake news detection, Natural Language Processing, Neural networks, Machine Learning, Security

## Abstract

Recent progress in the area of modern technologies confirms that information is not only a commodity but can also become a tool for competition and rivalry among governments and corporations, or can be applied by ill-willed people to use it in their hate speech practices. The impact of information is overpowering and can lead to many socially undesirable phenomena, such as panic or political instability. To eliminate the threats of fake news publishing, modern computer security systems need flexible and intelligent tools. The design of models meeting the above-mentioned criteria is enabled by artificial intelligence and, above all, by the state-of-the-art neural network architectures, applied in NLP tasks. The BERT neural network belongs to this type of architectures. This paper presents Transformer-based hybrid architectures applied to create models for detecting fake news.

## Introduction

The article is an extended version of the research work presented at the 13th International Conference on Computational Intelligence in Security for Information Systems CISIS 2020 [17, 19]. In the journal version of the article, a detailed description of the existing solutions in the area of fake news detection has been added; furthermore, an additional architecture (Fig. 2) is proposed, which is based on Transformers; the analysis and training of the neural network were extended with a dataset containing fake and real news from the COVID-19 area; a detailed description of the encoders included in BERT and RoBERTa has been added; additional charts related to the pre-experiment procedure, which were conducted to maximize metrics (Figs. 13, 14, 15, 16, 17), have been prepared.

Fake news is a growing plague affecting the political, social and economic life in many countries of the world. In an era when reliable information is a valuable resource, and when simultaneously there is the phenomenon of flooding of all kinds of information, the routines for filtering fake news are of particular importance in modern societies. It is expected that the issue of fake news will increase due to the emergence of 5G networks and thus increase the capacity and speed of the data transfer mechanisms, due to the increasing number of internet users, which will multiply the amount of information generated.

The sources of fake news can be websites, official websites of news agencies reporting to state institutions of competing countries or governments, and social media. In the era of the proliferation of devices and applications for transmitting data, even the fake news sent in the form of a private message can spread very quickly and cause, for example, the phenomenon of panic. Fake news is a powerful tool that can affect, for instance, the results of political elections or consumer shopping preferences. Therefore, it is crucial that state institutions and the bodies responsible for combating this type of abuse should have appropriate technical tools to detect fake news.

Fake news is generated by real authors, i.e., people, or more and more often by bots, that is virtual machines. The variety of natural language causes it that even relatively short textual information contains features characteristic of a given author or features characteristic of a given type of message. These features are relatively difficult to isolate, and only the analysis of a large material of a similar nature like fake news allows to extract them.

For texts analyzing and in the NLP (Natural Language Processing) tasks, the AI (Artificial Intelligence) algorithms and various types of deep learning methods are commonly applied [5,20, 18, 12, 4].

This paper focuses on designing the fake news detection model derived from the BERT (Bidirectional Encoder Representations from Transformers) architecture. It was decided to apply this method, since it is a relatively new solution, used in NLP since 2018 [7], which outperforms the existing static methods, such as GloVe (Global Vectors for Word Representation) in terms of the ability to detect context in the text. There have been some initial reports of the use of the BERT in detecting fake news.

For example, [30] describes the detection of government propaganda, [15] focuses on analyzing the title’s compliance with the contents of the text, and [27] conducts teaching on relatively small data to distinguish between fake news and satire. Attempts to combat fake news using machine learning and artificial intelligence techniques were also undertaken in other works using methods based on deep neural networks, on RNN recursive networks, or using traditional learnings algorithms like: RF (random forest), LR (logistic regression), NB (naive Bayes), MLP (multilayer perceptron) or support vector machine (SVM). There are a number of studies that compare older methods with the current SOTA (state-of-the-art) methods, using the older methods as a reference point. In [31], the authors compared 10 methods for detecting fake news in WeChat dataset. The presented results clearly indicate that the methods based on deep neural networks, such as CNN (convolutional neural network), LSTM (long short-term memory), EANN (event adversarial neural networks), detect fake news better than traditional methods such as LR, SVM or RF. In [31], with regard to fake news detection, the obtained f1-score result is 0.546 for the traditional SVM method, while for the EANN method this result is 0.731. In [8], the DTSL model, containing three CNN networks, was proposed for fake news detection. This model was compared with four traditional methods (naive Bayes, decision tree, Adaboost, support vector machine) and one method, based on deep neural networks, which is BRNN (bidirectional recurrent neural networks with LSTM). For DTSL, the f1-score was 0.6153, which significantly exceeded the results for the other tested methods: 0.1256 (SVM), 0.4124 (NB), 0.3585 (BRNN). A whole series of work related to disinformation and fake news detection emerged with the outbreak of the COVID-19 pandemic. In [10], a comparison was made of five methods (SVM, LR, RF, NB, MLP) detecting disinformation in relation to data related to the COVID-19 pandemic. For the f1-score the results ranged from 0.908 for the RF to 0.957 for the SVM [10]. In turn, the authors of the work [6] performed a comparative analysis of six Transformer- and DNN-based methods (XLNet, RoBERTa, XLMRoBERTa, DeBERT, ERNIE 2.0 and ELECTRA) to detect fake news. The obtained results show relatively small differences between the methods in relation to the f1-score, which ranges from 0.953 for the ELECTRA method (Efficiently Learning an Encoder that Classifies Token Replacements Accurately) to 0.972 for the RoBERTa method [6]. The authors improved the results using models ensemble, which allowed them to obtain the maximum f1-score of 0.9831 for the method RoBERTa+XLM-RoBERTa+XLNet+DeBERT. Another paper which concerned the detection of fake news related to the COVID-19 infodemics was [11], in which the XLNet with topic distribution method was proposed. The proposed method obtained an f1-score of 0.967, which exceeded all the other compared methods (USE + SVM, BERT with topic distributions, XLNet, Ensemble Approach: BERT and BERT + topic) [11].

In this paper, training was performed on data classified as true and false. Firstly, models for detecting fake news in article titles were created, followed by the models for detecting fake news in articles’ contents. High f1-scores were obtained for all models, which proves their reliability.

## BERT overview

The BERT is a modern architecture based on the transfer learning method. This method is defined as a breakthrough solution, gradually becoming the standard in the NLP; the solution displaces other methods previously used in performing typical NLP tasks such as sentiment analysis, Q&A (questions and answers), machine translation, classification, summarization or named entity recognition. The main distinguishing feature of the BERT method is its ability to recognize and capture the contextual meaning in a sentence or a text. This contextuality means that in the word embedding process, the numerical representation of a word or token depends on the surroundings of the word. This means that every time we take into account the surroundings of a word, the numerical value of the word will be different. This is a different approach to the word embeddings routine from the one for the static methods. Static word embeddings are coding unique words without taking their context into account, i.e., in the NLP methods based on this type of word embeddings, a unique word will always be coded in the same way. The GloVe method is also a static method; however, it exploits global statistical information to obtain word co-occurrences. Co-occurrences in the linguistic sense can be interpreted as an indicator of semantic proximity or an idiomatic expression. In contrary to the GloVe, the BERT belongs to a group of the methods other than static; we include it in the dynamic word embeddings methods. Dynamic methods are the methods that create language models that take the context mentioned above into account, but also the syntax and semantics of a text. The numerical representation of a unique word is not the same as in the static methods but depends on the word neighborhood and all the words (tokens) in the sentence (segment) of the text.

An important advantage and characteristic of the BERT is the application, similarly to the methods used in CV (computer vision) of the TL (transfer learning) principle [7]. The TL is based on the use of pre-trained models, which were created based on large datasets, and then on the use of the principle of fine-tuning. The fine-tuning leads to adapting the parameters of the architecture, previously trained to specific NLP tasks, for example to the Q & A tasks. In the BERT, there is the minimal difference between the pre-trained architecture and the final downstream architecture [7].

The abbreviation BERT stands for bidirectional encoder representations from transformers; the term Transformers refers to the network architecture that is based on Transformer blocks. The Transformer concept is based on replacing RNN (recurrent neural network) blocks in the neural network architecture with blocks using the self-attention mechanism. In the BERT, as the name suggests, Transformer encoders are used, and they are the only layer of the BERT architecture. For example, the BERT architecture with 12 layers contains 12 encoder blocks. The BERT is composed of a stack of identical layers. The number of the layers is the hyperparameter of the network. Each encoder block has two sublayers. The first is a multi-head self-attention mechanism, and the second is a simple, position-wise fully connected feed-forward network [29]. The self-attention is an attention mechanism relating different positions of a single sequence in order to compute a representation of the sequence [29]. The multi-head attention is a function responsible for calculations for many self-attention functions. In [29], a multi-head was defined as a mechanism that allows the model to jointly attend to information from different representation subspaces at different positions [29].

An important aspect of the BERT is its bidirectionality, which allows performing the analysis of tokens from right to left and from left to right of the examined text fragment. Such a model ensures training of the neural network taking the context of tokens into account. The BERT is designed to pre-train deep bidirectional representations from unlabeled text by jointly conditioning on both the left and right contexts in all the layers [7].

The BERT is seen as the leading method in NLP tasks related to the classification of texts and thus the classification of information and the detection of fake news in it. In the article [25], the results of the competition regarding the detection of fake news related to COVID-19 are presented. Despite the fact that participants used many different methods, the most successful models were BERT and its variations [25]. The advantages of the BERT over classic models, also called shallow learning (e.g., SVM, RF, LR, NB), should be sought in deep learning, which allows to learn feature representations directly from the input without too many manual interventions [21]. The BERT is an architecture containing self-attention layers; these layers allow for an attention mechanism to be applied. This is the advantage of the BERT over the CNN and the RNN methods, which are not intuitive enough for poor interpretability [21]. Also in the case of ELMO, which contains recursive LSTM layers, the advantage of the BERT in the tasks of text classification or hate speech detection is observed. This advantage should be collocated with the presence of Transformer architectures in the BERT, as it is presented in [9].

## Proposed application of the BERT

Since the creation of the first BERT architecture, subsequent modifications have been made, pre-trained on various datasets. The original BERT architectures are BERT$_{BASE}$ and BERT$_{LARGE}$; BERT$_{BASE}$ consists of: number of layers L=12, hidden size H=768, number of self-attention heads A=12, total parameters= 110M and BERT$_{LARGE}$ has the number of layers L=24, hidden size H=1024, number of self-attention heads A=16, total parameters=340M; from the originally created architectures, a whole series of their modified versions were created, such as the RoBERTa architecture, which has number of layers L=24, hidden size H=1024, number of self-attention heads A=16, total parameters= 355M. The RoBERTa was improved against the BERT through performance improvement [7]. The RoBERTa differs from the standard BERT by the improved pre-training procedure, which relied on training the model longer, with bigger batches over more data, removing the next sentence prediction objective, training on longer sequences and dynamically changing the masking pattern applied to the training data [23]. The RoBERTa architecture was proposed by the Facebook AI research team [23].

Another architecture developed with the BERT is DistilBERT, which includes the number of layers L=6, hidden size H=768, number of self-attention heads A=12, total parameters= 66M [7] [14]. The DistilBERT is a distilled version of the BERT, which was mainly created by reducing the number of layers. The creators of this architecture proved that it is possible to reach similar performances on many downstream tasks using much smaller language models pre-trained with knowledge distillation, resulting in models that are lighter and faster at inference time, while also requiring shorter computational training time [28].

All the mentioned architectures are available in the Flair library, which supports the Transformer-based architectures. The Flair is a tool that provides users with access to state-of-the-art methods in the NLP area. Many other models based on Transformers are available in Flair. The Flair allows users to choose the pre-trained architecture of the BERT, the description of which is available in the [14] documentation. The documentation contains described models dedicated to a single language (such as German), as well as multilingual models. The BERT model recommended in the Flair is ’bert-base-multilingual-cased’ that contains: the number of layers L=12, hidden size H=768, number of self-attention heads A=12, total parameters=110M, trained on cased text in the top 104 languages [14]. In Flair, all the types of embeddings are implemented with the use of the same interface [3].

**Fig. 1:**
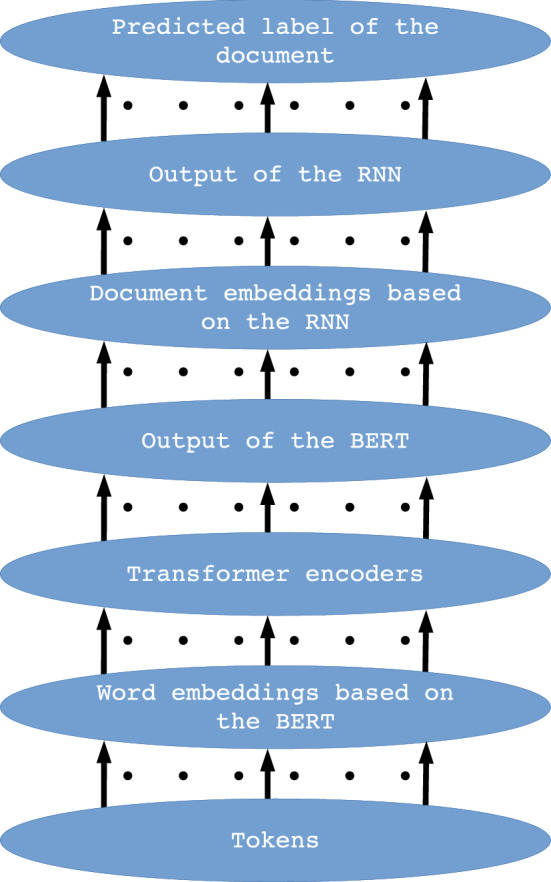
The proposed DRE hybrid architecture; the arrows represent streams of the data flow between the architecture layers. The number of streams depends on the number of input tokens

**Fig. 2:**
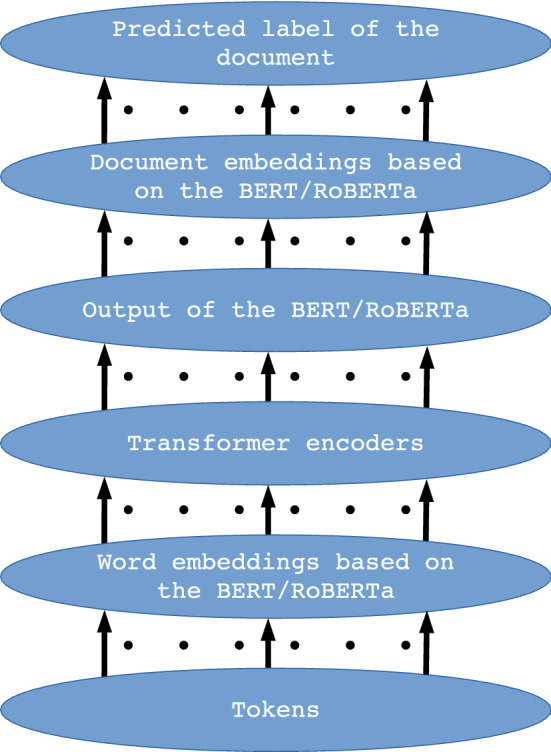
The proposed TDE hybrid architecture; the arrows represent streams of the data flow between the architecture layers. The number of streams depends on the number of input tokens

This paper uses selected BERT and BERT-derived methods available in the Flair library to create hybrid architectures. The models for detecting fake news have been trained through these architectures. Eight hybrid architectures were designed using pre-trained architectures. These hybrid architectures are the following: ’bert-base-multilingual-cased_DRE’, ’bert-base-cased_DRE’, ’bert-base-uncased_DRE’, ’bert-base-cased-finetuned-mrpc_DRE’, ’bert-large-uncased_TDE’, ’bert-large-uncased-whole-word-masking_TDE’, ’roberta-large_TDE’, ’roberta-large-openai-detector_TDE’. In the primary version of the article, general classes for words and documents embedding were used to create hybrid architectures. Due to the release of additional classes in the updated version of the Flair, additional designations have been introduced for hybrid architectures (_DRE and _TDE). The hybrid architecture is the result of three types of embedding classes, available in the Flair: CharacterEmbeddings, WordEmbeddings and DocumentEmbedding. The proposed method uses WordEmbeddings and the DocumentEmbedding class for word and document embeddings, respectively. In the hybrid architecture, the WordEmbeddings class is based on the BERT architecture and the DocumentEmbeddings class on the RNN architecture, or, in the second variant, the WordEmbeddings class is based on the Transformers (BERT /RoBERTa) architecture and the DocumentEmbeddings class on the pooler layer. Therefore, the paper presents two main variants of hybrid architectures: DRE (based on BERT and RNN) and TDE (based on BERT /RoBERTa and pooler layer). The DRE architectures are implemented by the DocumentRNNEmbeddings class and the TDE by the TransformerDocumentEmbeddings class. The TransformerDocumentEmbeddings class is a novelty tool in the Flair, implemented in 2020, and allowing the use of pre-trained transformers for all kind of embeddings (word-level, sentence-level and document-level embeddings) [2, 3]. The models of the proposed, main variants of the hybrid architectures are shown in Figs. 1, 2; Figure 2 presents the crucial differences between the two architectures. The four architectures created are the DRE-type architecture and four are the TDE-type architecture; all the architectures differ from each other in detail. Apart from the mentioned division of DRE, TDE, the discrepancies concern the number of layers, hidden states size and the value of hyperparameters, resulting from the training on different corpora of words. For the BERT architecture and related architectures, the maximum number of input tokens is 512. Although the DRE and the TDE apply the BERT and related architectures, these architectures implemented in the Flair differ in the maximum number of inputs. For the DRE, the maximum number of tokens for a single document was set to 100, and because the Transformer Document Embeddings class of the Flair library has a new feature that allows processing long sentences, the number of input tokens, for a single document in the TDE architecture is arbitrary. Both types of architectures (shown in Figs. 1, 2) contain the same output softmax layer, which indicates if the document was classified as fake, true, or as a data header. Probability of the classified label is also indicated in the top output layer. In the DRE architecture shown in Fig. 1, the data flow between the layers is as follows: up to 100 input tokens are entered into the “Word embeddings based on the BERT” layer, which generates the embedding vector (the number of generated vectors corresponds to the number of input tokens) for each token. Then, embedding vectors are passed to the “Transformer encoders” layers containing hidden layers the dimension of which is the same as the word embedding vector dimension and in the case of ’bert-base’ is 768. On the BERT output, we get the number of tensor representations according to the number of input tokens, and these tensors are the input for the “Document embeddings based on the RNN” layer, which will output a single embedding for the entire document, which contains up to 100 tokens. As the RNN is sequential, the maximum number of documents has not been specified for the DRE model. The trained RNN layer passes the built-in document representation to the next, top layer, where the classification takes place. The data flow for the architecture shown in Fig. 1 is similar to the data flow for the architecture in Fig. 2; however, for the TDE there is no strict limit of input tokens and document embeddings are based on the BERT. In the TDE, the data transferred between the layers also have different dimensions than in the DRE, which is related to the application of other versions of the Transformer in the TDE. The architectures shown in Fig. 1 and Fig. 2 are of a general nature. Below, a detailed description of the different versions of the BERT (in the DRE, ’bert-base’ versions were applied) and RoBERTa (in the TDE, ’bert-large’ and ’roberta-large’ versions were applied) was presented. The description is based on data obtained from the Flair tool.

**Fig. 3:**
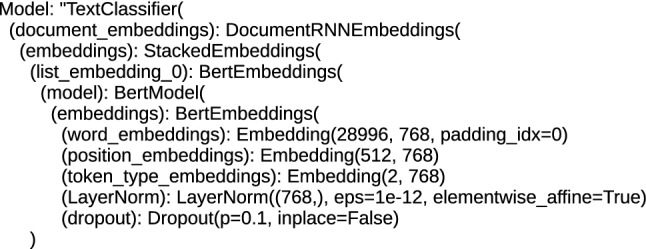
The initial structure of the DRE hybrid architecture

**Fig. 4:**
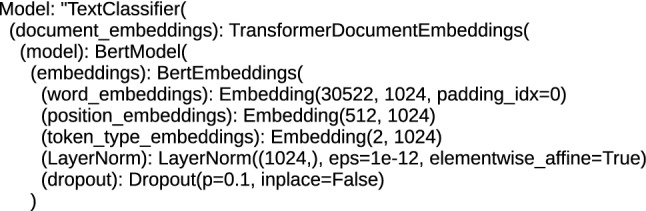
The initial structure of the TDE hybrid architecture

Each architecture, including BERT, applied to text processing, including the task of classification, performs tokenization and positioning of words in order to obtain the embeddings vector, which is a numerical representation of a word, character or token. This numerical form is the input for the bottom encoder of the BERT/RoBERTa. For the proposed architectures, the size of the word_embeddings vector ranges from (28996, 768) for the ’bert-base-cased-finetuned-mrpc_DRE’ and ’bert-base-cased_DRE’ architecture to (50265, 1024) for the ’roberta-large_TDE’ and ’roberta-large-openai-detector_TDE’ architecture. Position_embeddings vectors are from (512, 768) for the ’bert-base-uncased_DRE’, ’bert-base-multilingual-cased_DRE’, ’bert-base-cased_DRE’ and ’bert-base-cased-finetuned-mrpc_DRE’ architecture to (514, 1024) for the ’roberta-large_TDE’ and ’roberta-large-openai-detector_TDE’ architecture, the tokenization vector (token_type_embeddings) is (1, 1024) for ’roberta-large-openai-detector_TDE’ and ’roberta-large_TDE’, (2, 1024) for ’bert-large-uncased_TDE’ and ’bert-large-uncased-whole-word-masking_TDE’ and (2, 768) for ’bert-base-multilingual-cased_DRE’, ’bert-base-cased_DRE’, ’bert-base-uncased_DRE’ and ’bert-base-cased-finetuned-mrpc_DRE’. An example of the initial structure of the DocumentRNNEmbeddings class is shown in Fig. 3, and the initial part for the TransformerDocumentEmbeddings class is shown in Fig. 4. The structures presented in these figures are responsible for encoding the text input data into hidden state vectors.

**Fig. 5:**
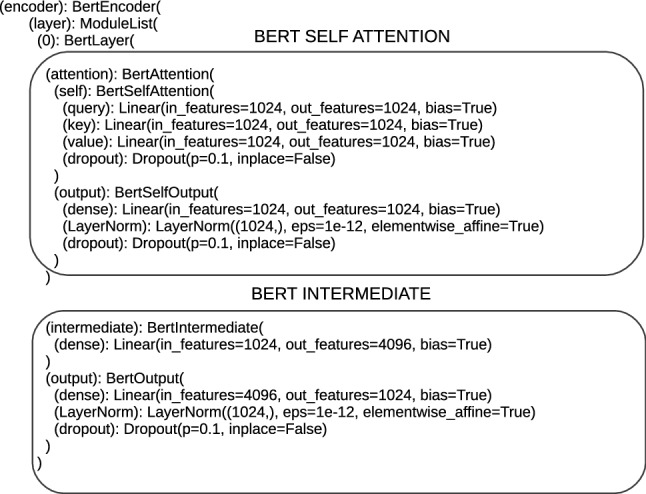
Single BERT encoder

**Fig. 6:**
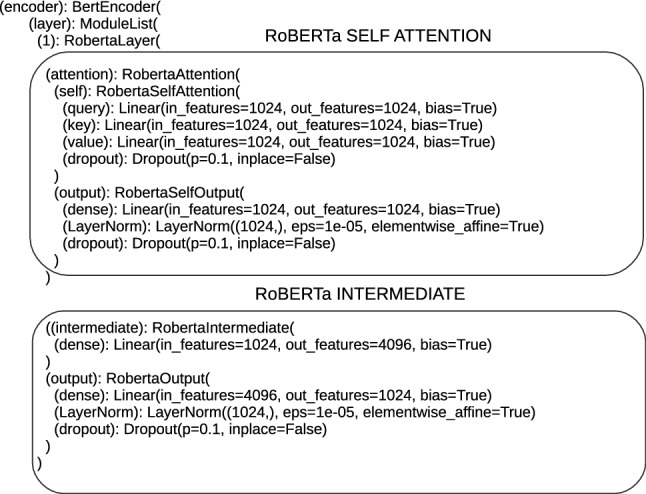
Single RoBERTa encoder

The next element of architectures is encoders; their number is not the same and amounts to 12 for ‘bert-base-multilingual-cased_DRE’, ‘bert-base-cased_DRE’, ‘bert-base-uncased_DRE’, ‘bert-base-cased-finetuned-mrpc_DRE’ and 24 for ‘bert-large-uncased_TDE’, ‘bert-large-uncased-whole-word-masking_TDE’, ‘roberta-large_TDE’, ‘roberta-large-openai-detector_TDE’. The number of encoders in architectures aligns with the number of Transformers layers. There are sublayers inside each encoder, at the bottom of the encoder, the first sublayer is the self-attention sublayer. The text is entered into the self-attention sublayer in numerical form, encoded in the embeddings vector. The self-attention sublayer is designed to calculate the weighted sum of the values, where the weight assigned to each value is computed by a compatibility function of the query with the corresponding key [29]. To calculate this value, the vectors Q (query), K (key), V (value) are required and they are defined in the architectures. The sizes of these vectors for the proposed architectures take the values 768 or 1024. In the self-attention sublayer (called BertSelfAttention or RobertaSelfAttention), a dropout operation is defined; the results of the attention mechanism are subject to. The following structures (RobertaSelfOutput and BertSelfOutput) relate to the operations performed before getting the output from the self-attention sublayer. These operations are add, layer-normalization and dropout again. The output of the self-attention is forwarded to feed-forward sublayers, which are marked in Flair, for proposed architectures as BertIntermediate or RobertaIntermediate. The data are carried through the dense layer, marked as (dense), which is a typical fully connected layer. Before being sent to the next encoder, this datum is subjected to add, layer-normalization and dropout operations. These operations are performed on the encoder output by means of the structure RobertaOutput or BertaOutput. Then, the data are passed through successive encoders with the same structure as described above. Single encoders are shown in Figs. 5, 6.

**Fig. 7:**
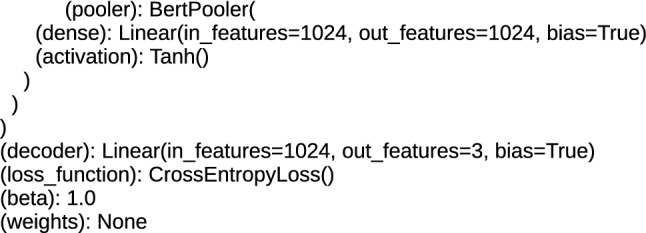
The top layers of the TDE, BERT architectures

**Fig. 8:**
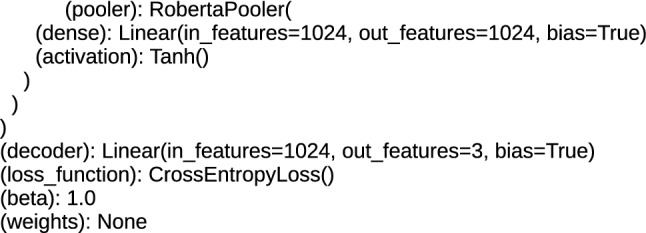
The top layers of the TDE, RoBERTa architectures

**Fig. 9:**
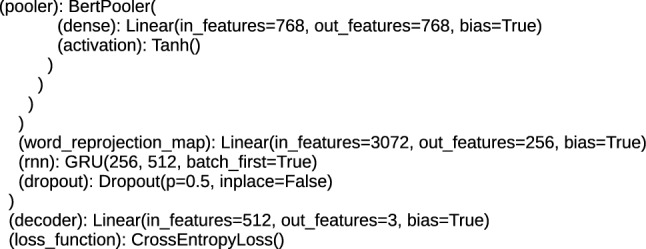
The top layers of the DRE, BERT architectures

Above the encoders, at the top of the architecture, there are layers for the task of text classification. These are additional layers applied in architectures, which are using pre-trained methods and prepared for the fine-tuning procedure. For DRE architectures, these layers are (rnn): GRU (gated recurrent units) and loss_function: CrossEntropyLoss, and for TDE it is the RobertaPooler or BertPooler layer, containing the dense sublayer and the activation function plus the structure for loss_function. RobertaPooler and BertPooler layers perform pooling over all word embeddings in the document. The rnn layer provides single embedding for the complete sentence [3]. Applied loss_function: CrossEntropyLoss computes the probability which ensures that the input data generated from the lower layers are classified. The top layers of the proposed architectures are presented in Figs. 7, 8 and 9.

The research was performed with the use of the remote Colaboratory Environment, the GPU card and the pandas tool, as well as the Python programming language. The applications of these tools and architectures are the paper’s novelty.

## Evaluation of the presented method

Several experiments were conducted to verify the usefulness of hybrid architectures in defense against fake news. They led to the following procedure: data selection, pre-processing of these data, selection of hyperparameters for the neural network training, training, verification of training process results, validation and testing, analysis of computational time needed to train the neural network.

### Data collections

The selection of appropriate data is a crucial element in neural network training. The work uses two datasets, the first one is available in ISOT (Information Security and Object Technology) research laboratory [1] and the second one is the COVID-19 dataset, which was prepared as part of the competition related to counteracting disinformation in the COVID-19 pandemic [26]. The ISOT dataset contains two files: one with fake news and the other with real (true) news. The dataset contains a total of 44898 items, 21417 of which are real items and 23481 are fake items. Each file contains four columns: article title, text, article publication date and the subject which can relate to one of six types of information (world news, politics news, government news, middle-east, US news, left news) [1]. The focus was on two columns, article ‘title’ and ‘text’, and the models were created for the data contained therein.

The COVID-19 dataset consists of three files that correspond to three sets for training, for validation [22] and for testing [16]. The sets contain classified data divided into two categories. For the sets training and validation, these categories are ’real’ and ’fake’, for the set test are ’0’ and ’1’, where ’0’ means false information and ’1’ means true. All the sets from the COVID-19 dataset are balanced in the amount of true and false information. Details describing the total number of messages in the sets and their quantitative breakdown by category are presented in Table 1

**Table 1:** Number of items in COVID-19 dataset [26]

Data	Total items	Fake (0)	Real (1)
Training set	6420	3060	3360
Validation set	2140	1020	1120
Test set	2140	1020	1120

The COVID-19 dataset was analyzed for the occurrence of emoticons, URL addresses and special characters, and they were observed in all three files (files). The frequency of the occurrence of particular words in the COVID-19 datasets was analyzed, and it was found that the definite article “the” is the most common, occurring 5960 times in the train set and 1994 times in the validation set. A large number of articles, conjunctions and pronouns in messages are natural and typical for natural language; therefore, the further part of the analysis concerned the occurrence of proper names. In this regard, a large number of occurrences of the words “COVID-19”, or with the hashtag “#COVID19”, the words “coronavirus” or “#coronavirus” were found. Tables 2 and 3 present the detailed number of occurrences of frequently occurring words, with particular emphasis on the occurrences of proper names. On the basis of this analysis, it was observed that the word “COVID” occurs frequently in all sets and in all the categories of these sets.

**Table 2:** Number of frequently occurring words in training set

Word/Token	Total	Occurrences in fake category	Occurrences in real category
the	5960	2475	3485
of	4537	1503	3034
to	3993	1543	2450
COVID-19	1191	644	547
#COVID19	1151	92	1059
coronavirus	640	582	58

**Table 3:** Number of frequently occurring words in validation set

Word/Token	Total	Occurrences in fake category	Occurrences in real category
the	1994	768	1226
of	1440	470	970
to	1292	469	823
COVID-19	377	193	184
#COVID19	357	31	326
coronavirus	203	183	20

The train and validation sets contain three columns, ’id’, ’tweet’ and ’label’, and the test set contains two columns ’label’ and ’text_a’.

Three data collections were prepared to design models and to execute the experiments. The collection 1 included an article ’title’ column and a new, added ’label’ column (with true and fake elements), and this collection was applied to detect fake news, based only on the article titles. This is a demanding task, in terms of model reliability, because titles are relatively short sentences, and with the NLP task of classifying texts, more reliable models are obtained by training the model on larger data. However, it is assumed that pre-trained BERT architectures allow designing reliable models for relatively small data sizes. The collection 2 contained ’text’ column and a new, added ’label’ column. This collection, in turn, was applied to create the fake news detection model based on the contents of the articles. The collection 3 is based on the COVID-19 dataset and includes two columns: ’text’, made up of the original ’tweet’ or ’text_a’ column, and a ’label’ column. The collection 3 was not limited in the number of tokens in any form and contains exactly the same number of tokens (words) as the original COVID-19 dataset.

### Data Pre-processing

To obtain essential information for the classification of texts, the elements that are repeated many times and are not a characteristic pattern for a given text are eliminated. It is dedicated to punctuation, periods, question marks, website addresses, links or e-mail addresses. All collections were subjected to the procedure of eliminating the above-mentioned elements in the texts. A large number of words being geographical or proper names were observed in the ISOT dataset in the ’text’ column of the true.csv file. These words had such a significant impact on the trained models that their presence or absence resulted in the classification of a given fragment of the text into the category of fake or true. Hence, the most repetitive words of this type were eliminated from the entire dataset. In the case of the COVID-19 datasets and the collection 3, the frequently repeated words were proper names such as “COVID” or “coronavirus”; hence, the pre-processing process eliminated these words. This is a different strategy than that adopted in the articles [13] and [22], where these words are also mentioned as frequently occurring, but they were not removed from the training data.

The data cleaned in such a way constituted the input for the training process.

### Experimental settings

Before the training, the architecture and hyperparameters were selected. In the Flair library, a user defines these elements by modifying the code. Different hyperparameters were adopted for the DRE architectures based experiments, for the TDE architecture-based experiments. To describe the DRE architecture-based experiments, two tables have been created. Table 4 presents the hyperparameters for the RNN part, and Table 5 presents the hyperparameters for the part of the hybrid architecture that is based on the BERT. Table 6 shows the hyperparameters for the experiments performed with the collection 3 and TDE architectures.

**Table 4:** Hyperparameter values of RNN

Name of the hyperparameter	Hyperparameter value
Learning rate	0.1
Batch size	32
Anneal factor	0.5
Patience	5
Max. number of epochs	5
Hidden states size	512

**Table 5:** Hyperparameter values of BERT

Name of the hyperparameter	Hyperparameter value
Learning rate	0.1
Batch size	32
Anneal factor	0.5
Patience	5
Max. number of epochs	5

**Table 6:** Hyperparameter values for experiments based on the collection 3 and TDE architectures

Name of the hyperparameter	Hyperparameter value
Learning rate	3e-5
Batch size	32
Anneal factor	0.5
Patience	3
Max. number of epochs	10

The collection 1 and the collection 2 were divided into training, testing and validation sets in the proportion 0.8 / 0.1 / 0.1, according to the cross-validation procedure. The COVID-19 dataset was already divided by its creators in the 0.6 / 0.2 / 0.2 ratio, and this division was maintained when performing the experiments related to the collection 3.

Due to the limitations of the BERT application in the Flair library version 0.4.5, the input strings were truncated to 100 tokens. Exceeding this limit led to excessive memory overload and interrupted the training process. Despite these limitations, the trained models correctly classified the news from external sources. Additional research work, consisting in performing experiments applying the COVID-19 dataset, was carried out using the Flair version 0.7, which did not require a reduction of the input strings.

### Results

In order to compare the models and demonstrate their reliability and usability, they were assessed by the following metrics: accuracy, precision, recall, f1-score, TP (true positive), TN (true negative), FP (false positive), FN (false negative). The results are presented in Table 7, which shows the results for the models trained on the collection 1, based on the ’title’ of articles; Table 8 presents the results for the models trained on the collection 2, based on ’text’ of the articles. Table 9 presents the results for the models trained on the collection 3, based on ’text’ (’tweet’) of the articles. The results obtained in the form of the accuracy above 90% testify to the reliability of the created models. Although no results were found in the literature for the ISOT dataset used in the article, for similar datasets and similar challenges related to the detecting fake news, the authors in articles [32] and [1] obtained results of a similar range. The COVID-19 dataset, related to the competition under CONSTRAINT 2021 [25] [26], was the subject of research in [13] [22] [16] primarily in the range of f1-score metrics. The best results obtained in this study, in relation to the f1-score, exceed all those reported so far. Also, for the remaining metrics, higher values were obtained than the ones previously reported.

**Table 7:** Resulted metrics for testing the models based on collection 1 (’title’) for the label fake (the comparison between architectures, ’bert-base-multilingual-cased_DRE’, ’bert-base-cased_DRE’, ’bert-base-uncased_DRE’, ’bert-base-cased-finetuned-mrpc_DRE’)

Metric	Bert-base	Base	’Base	’Finetuned
	-Multilingual	-Cased_DRE’	-Uncased_DRE’	-Mrpc_DRE’
	-Cased_DRE’			
True positive (TP)	2249	2346	2314	2346
True negative (TN)	2108	2139	2115	2139
False positive (FP)	35	4	28	4
False negative (FN)	28	1	33	1
Precision	0.9847	0.9983	0.9880	0.9983
Recall	0.9877	0.9996	0.9859	0.9996
f1-score	0.9862	0.9989	0.9870	0.9989
Accuracy	97.28%	99.89%	98.59%	99.89%

**Table 8:** Resulted metrics for testing the models based on collection 2 (’text’) for the label fake (the comparison between architectures, ’bert-base-multilingual-cased_DRE’, ’bert-base-cased_DRE’, ’bert-base-uncased_DRE’, ’bert-base-cased-finetuned-mrpc_DRE’)

Metric	’Bert-base	Base-cased_DRE’	Base	Finetuned
	-Multilingual		-Uncased_DRE’	-Mrpc_DRE’
	-Cased_DRE’			
True positive (TP)	2246	2271	2260	2213
True negative (TN)	2112	2087	2110	2107
False positive (FP)	31	56	33	36
False negative (FN)	31	6	17	64
Precision	0.9864	0.9759	0.9856	0.9840
Recall	0.9864	0.9974	0.9925	0.9719
f1-score	0.9864	0.9865	0.9890	0.9779
Accuracy	97.31%	97.34%	97.84%	95.68%

**Table 9:** Resulted metrics for testing the models based on collection 3 (’text’) for the label fake (the comparison between architectures, ’bert-large-uncased_TDE’, ’bert-large-uncased-whole-word-masking_TDE’, ’roberta-large_TDE’, ’roberta-large-openai-detector_TDE’)

Metric	Bert-large	Bert-large-uncased	Roberta	Roberta-large-
	-Uncased_TDE’	-Whole-word	-Large_TDE’	Openai-
		-Masking_TDE’		Detector_TDE’
True positive (TP)	1007	1005	1008	1011
True negative (TN)	1108	1105	1109	1104
False positive (FP)	12	15	11	16
False negative (FN)	13	15	12	9
Precision	0.9882	0.9853	0.9892	0.9844
Recall	0.9864	0.9853	0.9882	0.9912
f1-score	0.9877	0.9853	0.9887	0.9878
Accuracy	98.83%	98.60%	98.92%	98.83%

**Fig. 10:**
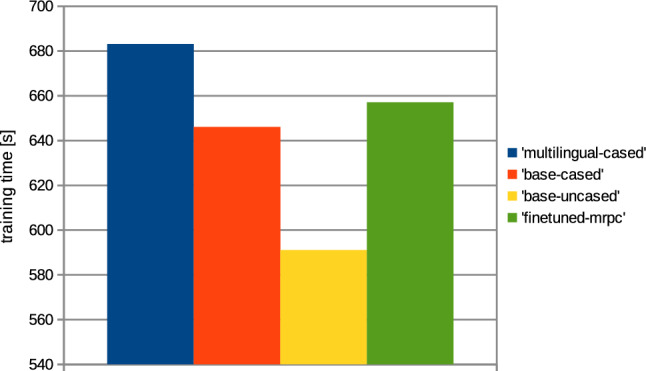
Computation time needed for model training, based on collection 1 (’title’); the comparison between various BERT architectures applied for the training

**Fig. 11:**
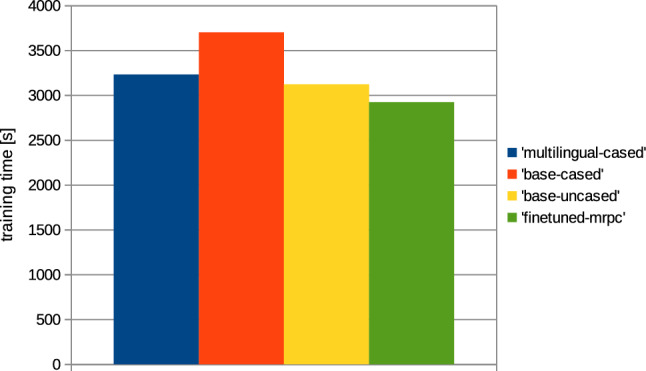
Computation time needed for models training, based on collection 2 (’text’); the comparison between various BERT architectures applied for the training

In addition to the metrics, the computation times required to train the network were also analyzed; the results are presented in Figs. 10 and 11.

**Fig. 12:**
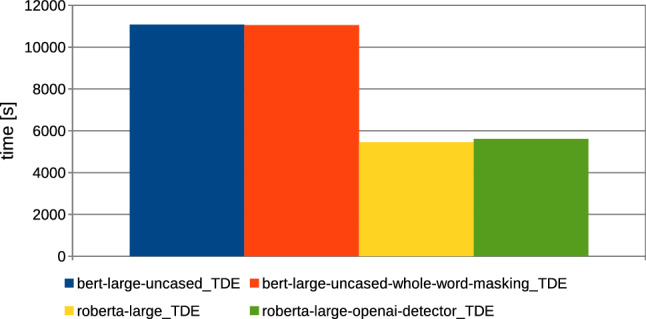
Computation time needed for models training, based on collection 3 (’text’); the comparison between various BERT/RoBERTa architectures applied for the training

### Results' refinement

The quality of the model expressed by relatively high metrics proves its validity and reliability. Various techniques are used to improve the quality of the metrics. The authors of the article [16], in order to maximize the f1-score metric, adopted, among others, a strategy of constructing handcrafted features that captured the statistical distribution of words and characters [16]. In turn, in the work [22] to maximize the metrics, pseudo-label algorithm and text-transformers architecture, consisting of five different Transformers models (BERT, Emie, RoBERTa, XL-net, Electra), were applied [22]. The BERT model applied was in the Covid-Twitter-Bert version, which is BERT large architecture pre-trained on large corpus of twitter messages dedicated to COVID topic [22] [24]. The pre-processing method was also applied to eliminate stop words and URLs. Pre-processing in the work [13] was also used to maximize the results; the procedure of removing or tokenizing hashtags, URLs, emoticons and mentions, changing the text to lowercase, eliminating punctuation and special characters was carried out [13]. In [13], the authors note that text processing can affect the quality of fake news detection; however, they do not clearly indicate which activities in the pre-processing routine maximize the results of the metrics obtained. In their best model, pre-processing was for URLs tokenization, emoticons converting into words, and text converting into lowercase. In [13], the authors experimented with various Transformers architectures, including BERT base and RoBERTa large; the best results, similar to [22], reported for models containing pre-trained Covid-Twitter-Bert architecture.

**Fig. 13:**
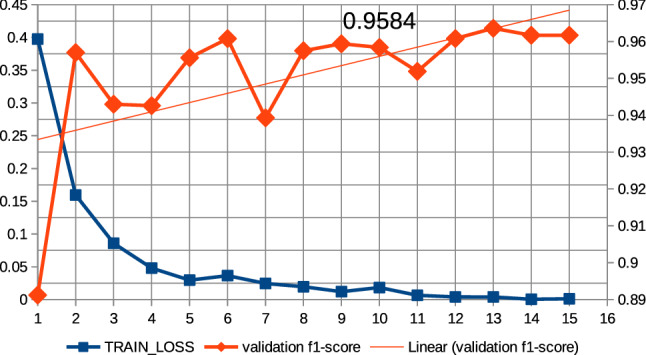
Relations between the number of epochs versus the f1-score for validation data and training loss for training data. The neural network training with the use of ’roberta-large_TDE’ architecture

**Fig. 14:**
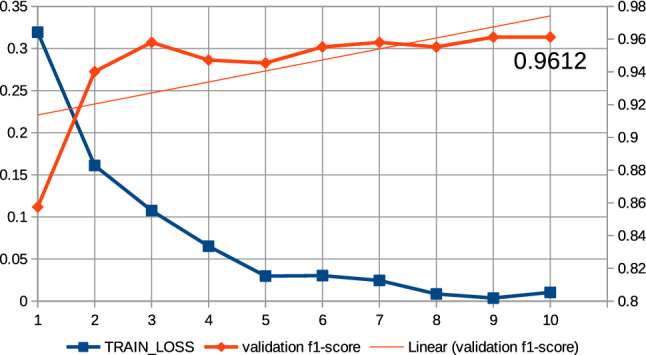
Relations between the number of epochs versus the f1-score for validation data and training loss for training data (collection 3). The neural network training with the use of ’roberta-large_TDE’ architecture and elimination from the text the most frequently words

**Fig. 15:**
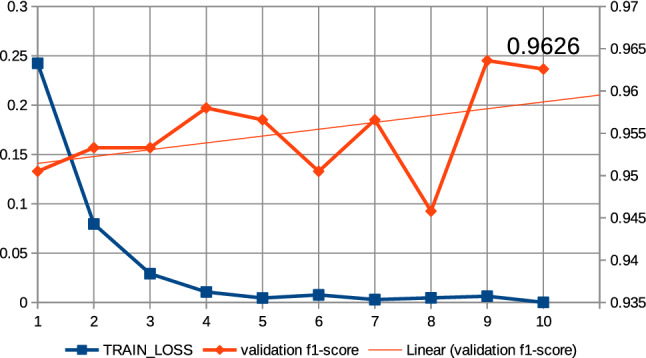
Relations between the number of epochs versus the f1-score for validation data and training loss for training data (collection 3). The neural network training with the use of ’bert-large-uncased_TDE’ architecture and elimination from the text the most frequently words

**Fig. 16:**
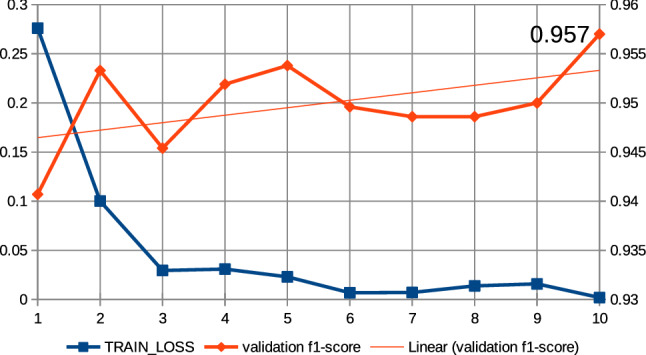
Relations between the number of epochs versus the f1-score for validation data and training loss for training data (collection 3). The neural network training with the use of ’bert-large-uncased-whole-word-masking_TDE’ architecture and elimination from the text the most frequently words

**Fig. 17:**
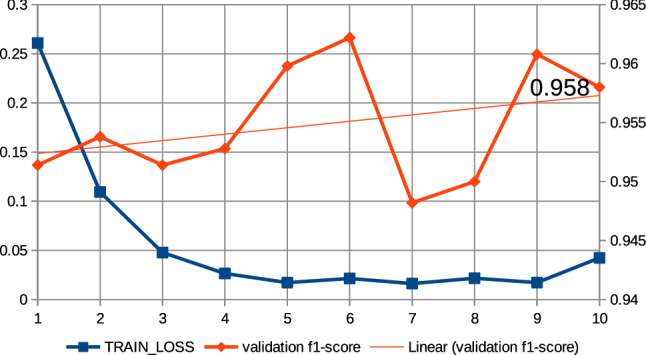
Relations between the number of epochs versus the f1-score for validation data and training loss for training data (collection 3). The neural network training with the use of ’roberta-large-openai-detector_TDE’ architecture and elimination from the text the most frequently words

In this work, a series of preliminary neural network training pre-experiments were conducted to maximize metrics. The first pre-experiment consisted in setting a relatively large number of epochs, amounting to 15, when training the neural network with the ’roberta-large_TDE’ architecture. On the basis of this pre-experiment, a common regularity was observed that the increase in training time, i.e., the increase in the number of epochs, increases the value of the metrics at the end of training and reduces losses. In this case, the f1-score was observed for validation data and the training loss was observed. It was also observed that above the 10th epoch the increase in the f1-score is relatively small; the results of the pre-experiment are shown in Fig. 13. Therefore, all subsequent models created on the basis of collection 3 were trained at hyperparameter max. number of epochs equal to 10. The next pre-experiment was to train the model with the ’robert-large_TDE’ architecture on the data, of which the most common words such as “COVID”, “COVID19”, “coronavirus” were eliminated. As shown in Figs 13, 14, for epoch 10 a better f1-score is obtained for validation data when the most common words are reduced. The two techniques mentioned above were applied to train all models, which are based during the training routine on collection 3. Figures 15, 16 and 17 show the training loss and validation f1-score obtained during the model training procedure with the use of collection 3. These charts confirm that there is an increasing correlation between the epoch number and the f1-score value for validation data.

The study obtained the best result in relation to the f1-score equal to 0.9887 for the dataset test, using the ’roberta-large_TDE’ architecture based on pre-trained RoBERTa large. The RoBERT large was also subject of experiment in the paper [13]; the f1-score equal to 0.9762 was obtained. The discrepancy in the results, despite the application of a similar architecture, should be explained in the shorter learning time of the model; the authors used three epochs and a different pre-processing strategy, the most common words were not eliminated, as in this paper. For the ’bert-large-uncased_TDE’ architecture proposed in this paper, in the testing phase for the COVID-19 test dataset, the f1-score was 0.9877, which outperforms the results of the BERT large (Covid-Twitter-Bert) experiments proposed in [13] and [22]. In the case of [13], the discrepancy is due to a small number of epochs and a different pre-processing strategy, while in the case of the Covid-Twitter-Bert model from [22], although the authors used 12 epochs, the Covid-Twitter-Bert is only one of the five models in architecture and therefore its influence on the final result is much smaller than the influence of BERT large in the ’bert-large-uncased_TDE’ architecture proposed in this work.

The strategies for maximizing the results of metrics proposed in this paper head to extending the training process and the maximum reduction of text in the pre-processing process. The adopted strategies have resulted in models that outperform the results reported so far.

## Conclusion

In this paper, the procedures for creating the models for detecting fake news using the hybrid architecture were presented. These architectures are mostly based on various types of pre-trained embeddings of the BERT for word embeddings and on the RNN or on the BERT/ RoBERTa pooler for document embeddings. The procedures related to the network training and the data preparation for training were carried out using the remote platform and the GPU card available there. The procedures applied for creating the models are the paper’s novelty. The careful analysis indicated that the application of the Flair-based hybrid architecture and simultaneously the TransformerDocumentEmbeddings class for fake news detection tasks has not been reported in the literature yet.

The BERT technique and its modifications have a crucial impact on the NLP, and the BERT is still a relevant research topic for artificial intelligence scientists. In the paper, we presented valid, robust models, which are based on the state-of-the-art methods, derived from the Flair library. The models are the scientific contribution of this paper to the NLP research domain.

The designed models are solid and reliable, ready to use in real-time fake news detection systems.

Our future work is focused on transfer learning where models can be used in various domains and re-trained effectively while being used.
